# Biofilm-associated bacterial amyloids dampen inflammation in the gut: oral treatment with curli fibres reduces the severity of hapten-induced colitis in mice

**DOI:** 10.1038/npjbiofilms.2015.19

**Published:** 2015-10-14

**Authors:** Gertrude O Oppong, Glenn J Rapsinski, Sarah A Tursi, Steven G Biesecker, Andres J P Klein-Szanto, Mark Goulian, Christine McCauley, Catherine Healy, R Paul Wilson, Cagla Tükel

**Affiliations:** 1 Department of Microbiology and Immunology, School of Medicine, Temple University, Philadelphia, PA, USA; 2 Department of Pathology, Fox Chase Cancer Center, Philadelphia, PA, USA; 3 Department of Biology, University of Pennsylvania, Philadelphia, PA, USA; 4 Janssen Research & Development, LLC, Spring House, PA, USA

## Abstract

**Background/objectives:**

A disruption of epithelial barrier function can lead to intestinal inflammation. Toll-like receptor (TLR) 2 activation by microbial products promotes intestinal epithelial integrity and overall gut health. Several bacterial species, including enteric bacteria, actively produce amyloid proteins as a part of their biofilms. Recognition of amyloid fibres found in enteric biofilms, termed curli, by the Toll-like receptor (TLR)2/1 complex reinforces barrier function. Here, we investigated the effect of purified curli fibres on inflammation in a mouse model of acute colitis.

**Methods:**

Bone marrow–derived macrophages as well as lamina propria cells were treated with curli fibres of both pathogenic *Salmonella enterica* serovar Typhimurium and commensal *Escherichia coli* Nissle 1917 biofilms. Mice were given 0.1 or 0.4 mg of purified curli orally 1 day post administration of 1% 2,4,6-trinitrobenzene sulphonic acid (TNBS) enema. Histopathological analysis was performed on distal colonic tissue taken 6 days post TNBS enema. RNA extracted from colonic tissue was subjected to RT-PCR.

**Results:**

Here we show that curli fibres of both pathogenic and commensal bacteria are recognised by TLR2 leading to the production of IL-10, immunomodulatory cytokine of intestinal homeostasis. Treatment of mice with a single dose of curli heightens transcript levels of *Il10* in the colon and ameliorates the disease pathology in TNBS-induced colitis. Curli treatment is comparable to the treatment with anti-tumour necrosis factor alpha (anti-TNFα) antibodies, a treatment known to reduce the severity of acute colitis in humans and mice.

**Conclusion:**

These results suggest that the bacterial amyloids had a role in helping to maintain immune homeostasis in the intestinal mucosa via the TLR2/IL-10 axis. Furthermore, bacterial amyloids may be a potential candidate therapeutic to treat intestinal inflammatory disorders owing to their remarkable immunomodulatory activity.

## Introduction

The human gastrointestinal (GI) immune system encounters an estimated one hundred trillion bacteria representing more than 1,000 species.^1–3^ A large percentage of these populations of bacteria live in the distal GI tract.^4^ Once thought of as passive participants in GI homeostasis, it is now known that the microbiota are actively involved in initiating immune responses that contribute to GI immune cell development and homeostasis. Recognition of gut microbiota and microbiota-derived components via pattern recognition receptors (PRRs) help maintain mucosal barrier integrity. Toll-like receptor (TLR) 2 has been widely discussed in the literature to have a pivotal role not just in maintaining intestinal epithelial integrity but also in mediating immune responses that promote overall gut health.^5–8^ TLR2-deficient mice are more susceptible to bacteria-induced^9^ and chemically induced colitis.^6^ Moreover, TLR2 recognition of commensal bacteria is required for intestinal homeostasis.^8^ TLR2 signalling depends on the adaptor molecules MyD88 or TIRAP/MAL resulting in a pro- or anti-inflammatory outcome.^5,10,11^ In the intestinal mucosa, TLR2 mostly regulates the anti-inflammatory responses and the reinforcement of the epithelial barrier. Recruitment of the PI3K-Akt to the MyD88/MAL complex upon TLR2 activation results in the expression of anti-inflammatory cytokines including IL-10 and repression of the pro-inflammatory pathway.^12^ Furthermore, activation of PI3K-Akt leads to the expression of tight junction proteins such as ZO-1 in epithelial cells.^6,7^ IL-10 has been implicated to have a vital role in maintaining gut homeostasis. In fact, IL-10-deficient mice raised in conventional animal housing develop spontaneous enterocolitis by 4–8 weeks of age.^13^

Lipoproteins from Gram-negative bacteria are canonical TLR2/1 ligands.^14^ However, lipoproteins are buried in the outer membrane and stimulation of the TLR2/1 receptor complex by intact *E. coli* cells is dominated by curli,^15^ an amyloid secreted to the extracellular matrix of biofilm by both commensal and pathogenic members of the *Enterobacteriaceae*.^16–19^ Curli fibres, initially described in *S.* Typhimurium as thin aggregative fimbriae, are encoded by the *csg* gene cluster that consist of *csgBAC* and *csgDEFG* genes.^20^ The major subunit of curli, CsgA, is transcribed from the *csgA gene* under the control of the master regulator CsgD.^21^ Although CsgA has the intrinsic propensity to self-aggregate, it requires the CsgB subunit for nucleation into fibres.^22–24^ Curli-like proteins have been found within four phyla; Bacteroidetes, Proteobacteria, Firmicutes and Thermodesulfobacteria.^18,25^ Curli are an essential proteinaceous component of the extracellular biofilm matrix that allows enteric bacteria to bind to biotic and abiotic surfaces.^26–28^ We recently determined that the innate immune recognition of curli is mediated by TLR2/1 heterocomplex.^15,29,30^ Recognition of curli fibres by TLR2 complex leads to the augmentation of the intestinal epithelial barrier and limits bacterial translocation from the intestinal lumen during infection.^31^ In addition, the NLRP3 inflammasome cooperates with the TLR2/1 receptor complex to recognise curli fibres leading to the production of IL-1β.^32^ Interestingly, both TLR2 and NLRP3 have been implicated for the recognition of human amyloids. In addition to the latter two receptors, recently formyl peptide receptor 2 was identified as a receptor for human amyloids, amyloid beta and serum amyloid A, and the bacterial amyloid, phenol soluble modulins (PSM) of *Staphylococcus aureus*.^33–36^

Curli fibres possess a cross β-sheet quaternary structure that is characteristic of amyloids in general and makes these proteins resistant to proteolytic digestion,^18,20,37^ a property that should facilitate intact transition through the GI tract. We thus investigated whether oral administration of this TLR2 ligand would ameliorate inflammation in a mouse model of colitis.

## Materials and Methods

### Bacterial strains and culture conditions

*E. coli* Nissle 1917 (EcN) was first described by physician Alfred Nissle.^38^ *Salmonella enterica* serovar Typhimurium (denoted as STM) *msbB* mutant (RPW3) was described previously.^39^ Bacterial growth was supported with 50 μg/ml of kanamycin or 100 μg/ml nalidixic acid where needed.

### Purification of curli fibres

To induce the production of curli fibres, 500 μl of the overnight culture of bacteria was inoculated into 200 ml of YESCA broth supplemented with 4% dimethylsulphoxide (ACROS) at 28 °C for 72 h with shaking at 200 r.p.m.^40^ Purification of curli fibres from the *S*. Typhimurium *msbB* mutant (RPW3) or EcN was performed according to an established protocol.^41^ Briefly, bacterial cells were collected and subjected to multiple treatments of lyzozyme, DNAse and RNAse as well as boiling in SDS. Samples were run overnight in a 12% SDS-preparative gel and insoluble curli fibres were recovered from the well.

### Cell/tissue culture

#### Bone marrow–derived macrophages

Bone marrow–derived macrophages were generated from age-matched C57BL/6 or TLR2−/− (approximately 6–8 weeks) female mice as described previously.^29^ Supernatants were assayed for IL-10 by ELISA (eBioscience, Hatfield, UK). Experiments were repeated three times.

#### Lamina propria cells

Lamina propria (LP) cells were isolated using the Lamina Propria Dissociation Kit (mouse) from Miltenyi Biotec (Bergisch Gladbach, Germany). The LP cells were processed from whole-mouse small and large intestine using the company’s suggested protocol. The experiments were repeated three times.

#### Transfection of HeLa cells

Transfection studies using HeLa cells and the vectors were described previously.^15^ The cells were stimulated with 2.5 μg/ml purified curli, GST, GST–CsgA or Pam_3_CSK_4_ (Invivogen, San Diego, CA, USA).

### Experimental colitis

The 2,4,6-trinitrobenzenesulfonic acid (TNBS)-induced colitis model is a well-characterized animal model of inflammatory bowel disease.^42^ Briefly, groups of six to seven 4–6-week Balb/cJ mice were starved 24 h before receiving an enema containing 60 μl of 50% ethanol solution (mock) or a 1% TNBS solution in 50% ethanol intrarectally approximately 3-cm into the colon. A day after TNBS colitis induction, mice were given the following treatments (per mouse): 0.1 or 0.4 mg curli fibres in 100 μl of water or 100 μl sterile water as a mock via oral gavage or 100 μg of anti-tumour necrosis factor alpha (anti-TNFα) via intraperitoneal injection as a positive control. Animals were monitored daily for weight/physical changes for 3 days or 6 days. On day 3 or 6 post TNBS induction, mice were euthanized via carbon dioxide asphyxiation. Samples from the colon were collected for messenger RNA and histopathology analysis. Colitis experiments were repeated three times. Sample size for this experiment was chosen to obtain statistical significance between groups on the basis of statistical power calculations (*n*=2SD^2^(Z^−3^+Z)^2^/d^2^), previous experience and literature review. No randomisation was used. Colitis experiments were approved by Temple University’s Institutional Animal Care and Use Committee.

### Histopathology

Tissue samples from colon were harvested immediately following euthanasia and were fixed with 10% buffered formalin. Colon tissue samples were cut and stained with hematoxylin and eosin at the Fox Chase Cancer Center Histopathology Core Facility. Colitis was scored independently and blindly by a pathologist.

### Real-time PCR

To examine messenger RNA levels of *Il-10*, *Tgf-β*,^43^ *Ifn-γ*,^44^ *Tnfα*^45^ and *Il-6*,^44^ tissue samples from mice were taken at the time of euthanasia and immediately frozen in liquid nitrogen for further processing. RNA was isolated from the tissue using the Qiagen RNeasy kit according to company specifications. Following RNA isolation, 1 μg of RNA was reverse transcribed using murine leukaemia virus reverse transcriptase (Applied Biosciences). Complementary DNA (2.5 μl) was subjected to quantitative PCR amplification using SYBR Green (Applied Biosystems, Waltham, MA, USA) and the primers listed above. Fold change differences in amplification were determined using the ΔC_T_ approach with *Gapdh*^46^ as the housekeeping gene.

### Statistical analysis

Differences between data sets were analysed using the Student’s *t*-test (*P* value <0.05 is significant) where applicable. For the analysis of bacterial numbers, values were logarithmically converted before statistical analysis. Histopathological scores were analysed using the Mann–Whitney test.

## Results

### Il-10 is induced in bone marrow–derived macrophages in response to curli fibres in a TLR2-dependent manner

**Figure 1:**
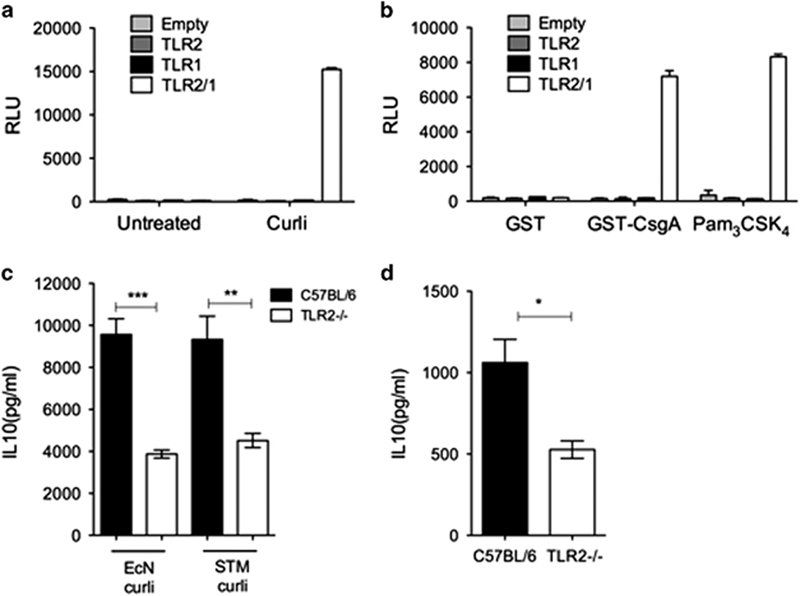
Curli fibres from a commensal and pathogen induce cytokines from bone marrow–derived macrophages in a TLR2-dependent manner. HeLa cells carrying a NFkB luciferase reporter were mock-transfected (vector) or transfected with TLR1, TLR2 or TLR2/TLR1. Cells were stimulated with (**a**) 2.5 μg/ml purified curli or (**b**) GST–CsgA fusion protein or synthetic tri-acylated lipopeptide (Pam_3_CSK_4_). Untreated cells or cells stimulated with GST protein served as negative controls. Activity of the NFκB luciferase reporter activity was measured and expressed as relative luminescence units (RLU). (**c**) Bone marrow–derived macrophages (BMDMs) from 6 to 8-week-old female C57BL/6 (*n*=3) and TLR2−/− (*n*=3) old mice were treated with 5 μg/ml of curli fibres isolated from biofilms of STM or EcN for 24 h. Supernatants were collected and IL-10 protein levels were measured using ELISA. IL-10 was not detected in samples that were unstimulated (control). (**d**) Lamina propria (LP) cells were isolated from the intestine of C57BL/6 (*n*=3) and TLR2−/− (*n*=3) mice as described in the Materials and Methods. LP cells were incubated with 5 μg/ml curli fibres for 24 h. Supernatants were then collected and assayed for IL-10 by ELISA. (*P value <0.05; 95% confidence interval). STM, *Salmonella enterica* serovar Typhimurium. ELISA, enzyme-linked immunosorbent assay.

Curli fibres are produced by commensal and pathogenic strains of *Enterobacteriaceae* family members including *E. coli* and *S.* Typhimurium. Our previous studies have established that curli fibres from *S.* Typhimurium are recognised by TLR2 (refs 15,29,30,47,^48^) and specifically the TLR2/1 heterocomplex recognises both the purified curli as well as the recombinant GST-tagged CsgA (Figures 1a and b). Previous studies had shown that a commensal strain of *E. coli* Nissle 1917 (EcN), dampens inflammation through the production of IL-10 via TLR2 activation in the GI tract.^49,50^ Triacylated lipoproteins from Gram-negative bacteria are canonical TLR2/1 ligands (Supplementary Figure S1). However, as EcN also expresses curli fibres (Supplementary Figure S2), the mechanism by which the EcN triggers TLR2 activation in the gut remains unknown. Thus, we first wanted to compare the stimulatory activities of curli fibres from pathogenic vs commensal bacteria and determine whether curli fibres induced IL-10 production in immune cells. We used TLR2−/− mice that are on C57BL/6 mice background. Stimulation with curli fibres purified from EcN elicited induction of IL-10 in bone marrow–derived macrophages from C57BL/6 mice, whereas this response was blunted in macrophages derived from TLR2−/− mice (Figure 1c). Similar results were obtained when the experiment was repeated with curli purified from an *S*. Typhimurium *msbB* mutant, which expresses a modified lipid A that does not signal through TLR4,^51^ suggesting that LPS did not markedly contribute to the observed responses. Furthermore, macrophages produced similar levels of IL-6 in response to curli fibres from both species in a TLR2-dependent manner (Supplementary Figure S3).

As TLR2 has been reported previously to be an essential receptor for GI homeostasis and repair,^5–7,9^ we investigated the interaction of curli fibres with cells of the GI tract by treating the intestinal LP cells isolated from the colonic tissue of C57BL/6 mice and TLR2−/− mice with purified curli fibres. Consistent with what we had noted with bone marrow–derived macrophages, *ex vivo* treatment of intestinal cells with curli fibres resulted in the production of IL-10 in a TLR2-dependent manner (Figure 1d). Overall, these results indicated that curli fibres are recognised by TLR2, which results in the production of IL-10.

### Oral administration of curli fibres in non-colitic mice does not elicit cytokine production

**Figure 2:**
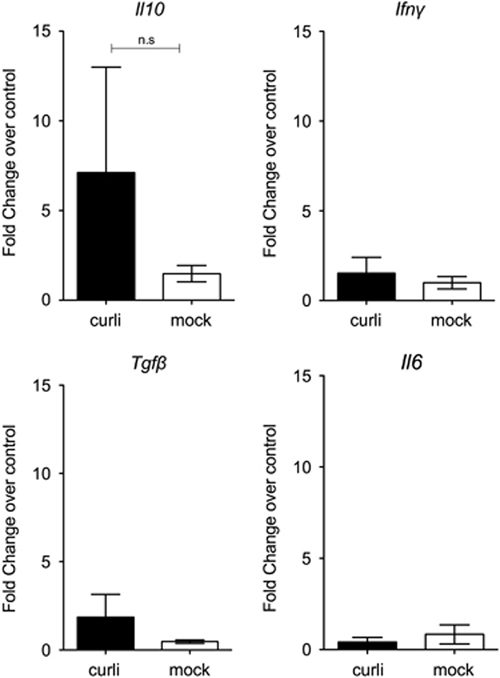
Oral inoculation of curli fibres into mice pretreated with antibiotics did not elicit cytokine expression in the intestine. Female C57BL/6 mice (*n*=5) were given 0.1 mg of curli fibres orally following a 7-day treatment of mice with a broad-spectrum antibiotic cocktail. Colonic samples were harvested and subjected to RNA extraction after 72 h. Real-time PCR for *Il10*, *Ifn-γ*, *Tgf-β* and *Il6* was performed on mRNA (messenger RNA).

The β-sheet structure of amyloids makes them resistant to chemical as well as enzymatic treatments. In fact, amyloids only dissociate into their monomers by treatment with 90% formic acid or hexafluoroisopropanol treatment *in vitro*.^52^ Thus, amyloids may be resilient to conditions encountered in the intestinal lumen. Consistent with this idea, curli fibres can be detected in faecal matter for up to 12 h after oral administration (Supplementary Figure S4). To test the effect of curli fibres on mucosal gene expression in the intestine, we treated C57BL/6 mice with a cocktail of antibiotics including ampicillin (1 g/l), neomycin (1 g/l), metronidazole (1 g/l) and vancomycin (0.5 g/l) for 7 days in drinking water to mimic germ-free conditions.^53^ Following intensive antibiotic administration, 0.1 mg curli fibres were administered via oral gavage and cytokine production was determined in the intestinal tissue from these mice using quantitative RT-PCR. There were no significant changes in *Il10*, *Ifng*, *Tgfb* or *Il6* messenger RNA expression between the treatment and control groups 72 h post administration (Figure 2) suggesting that curli fibres do not elicit significant changes in cytokine expression during steady-state conditions in the gut.

### Intraperitoneal injection of curli fibres induces interleukin-10 expression in mouse small intestinal tissue

**Figure 3:**
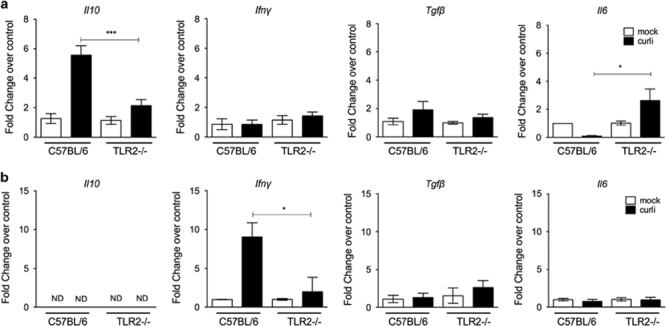
Intraperitoneal injection of curli elicits *Il10* expression in intestinal tissue of mice in a TLR2-dependent manner. Age-matched female C57BL/6 and TLR2−/− mice (*n*=5) were injected intraperitoneally with 0.1 mg of curli fibres. After 72 h, real-time PCR was performed on intestine (**a**) and spleen (**b**) for *Il10*, *Ifn-γ*, *Tgf-β* and *Il6*. (*P<0.05; ***P<0.001).

Systemic administration of flagellin induces changes in gene expression in intestinal epithelial cells and Paneth cells along the entire length of the small intestine.^54^ As the administration of curli fibres via oral gavage of mice with an intact epithelial barrier did not elicit detectable immune responses (Figure 2), we wanted to investigate whether the immune cells underlying the LP have an important role in responses to curli. We thus injected C57BL/6 or TLR2−/− mice intraperitoneally with the curli fibres. Seventy-two hours after injection, we determined the gene expression profiles in the small intestine and spleen by quantitative RT-PCR. Interestingly, *Il10* expression was upregulated in the small intestinal tissue from C57BL/6 mice at 72 h. Remarkably, the expression of *Il10* was blunted significantly in TLR2−/− mice. No upregulation of pro-inflammatory cytokines *Ifng, Il6* or another immunomodulatory cytokine *Tgfb* was observed in the small intestinal tissue (Figure 3a). Intriguingly, a completely different pattern of cytokine expression was observed in the spleen. Although we did not detect *Il10*, *Tgfb* or *Il6*, transcripts for *Ifng* were induced by curli in a TLR2-dependent manner (Figure 3b). These data suggested that (i) the subepithelial LP cells have an important role in eliciting IL-10 production in response to curli fibres and (ii) the immune response against curli fibres in the gut is different from that encountered at systemic sites such as the spleen possibly owing to the presence of specialized immune cell populations previously reported in the gut.^55–57^

### One time administration of curli fibres ameliorates the severity of TNBS colitis

**Figure 4:**
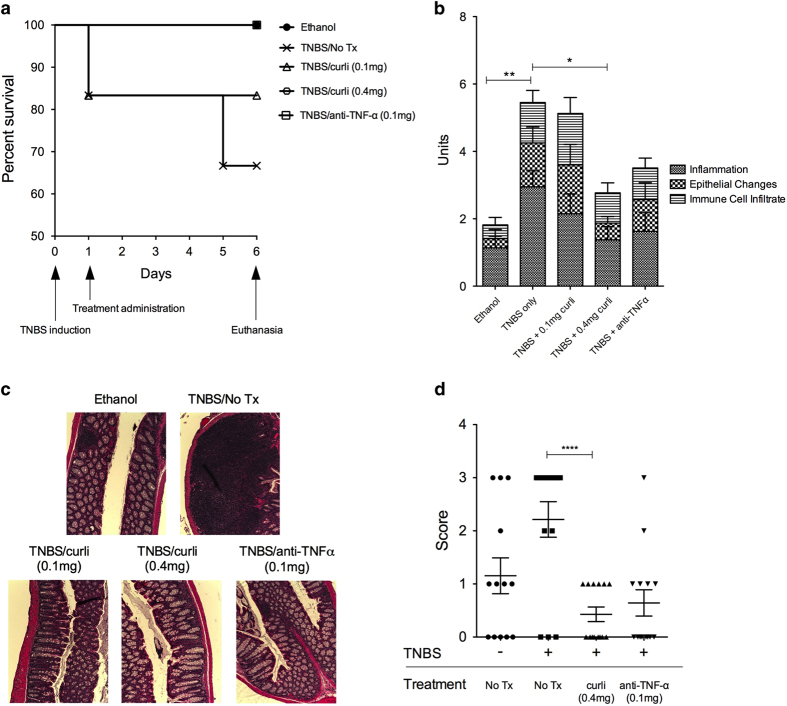
Single treatment with curli fibres ameliorates TNBS colitis. Colitis was induced in 6–8-week female Balb/c (*n*=6–7) mice by intrarectal instillation of 1% TNBS in 50% ethanol or 50% ethanol as a vehicle control. Day 1 post TNBS enema, mice were administered treatments as follows: 0.1 mg curli (oral), 0.4 mg curli (oral), 0.1 mg anti-TNFα (i.p.) or no treatment. (**a**) survival (*n*=6), (**b**) histopathological scores at day 6 post TNBS induction were plotted and (**c**) H&E images were taken. (**d**) Stool consistency scores were determined at day 3 post TNBS induction. It should be noted that larger areas of immune cell infiltration and lymphoid follicles in the submucosa was determined in the colonic tissue of mice treated with TNBS alone as compared with the groups that received curli treatment (*P<0.05; **P<0.01; ****P<0.0001). H&E, hematoxylin and eosin; i.p., intraperitoneal; TNBS, 2,4,6-trinitrobenzene sulphonic acid; TNF, tumour necrosis factor; Tx, treatment.

Next, we investigated whether oral administration of curli altered mucosal responses in the setting of severe colitis, a condition during which epithelial erosion damages barrier function. We chose TNBS-induced colitis model, an experimental colitis induced in susceptible strains of mice. Although Balb/c, SJL mice are susceptible to TNBS-induced colitis, C57/BL6 mice are resistant and do not develop colitis.^42^ Groups of six to seven Balb/c mice were administered intrarectal instillations of 60 μl of 1% TNBS in 50% ethanol (EtOH) to induce colitis or received vehicle control (intrarectal instillations of 60 μl of 50% EtOH). One day after TNBS administration, mice were mock-treated or treated intragastrically with 0.1 or 0.4 mg purified curli fibres. We compared these groups of mice with another group of mice with TNBS colitis that received an intraperitoneal injection of 0.1 mg of anti-TNFα antibodies as a control, a treatment known to reduce the severity of TNBS colitis.^58^ Mice were euthanized on day 6 after TNBS treatments (Supplementary Figure S5). Weight changes were monitored throughout the experiment (Supplementary Figure S6). Although the mice that received only TNBS enema had a survival rate of 65%, mice that received 0.1 mg curli fibres intragastrically had an 80% survival rate. Hundred percent mice that received 0.4 mg of curli fibres survived the treatment (Figure 4a). When colonic tissues were scored histopathologically for inflammatory changes, no differences were observed between groups at 2 days after treatments (data not shown). At day 6 post treatment, pathology scores for mice that received 0.1 mg curli fibres intragastrically 1 day post TNBS enema did not differ significantly from the mice that only received TNBS enema. However, single-dose intragastric treatment with 0.4 mg curli fibres 1 day post TNBS enema decreased the pathology at day 6 and the scores were comparable to the control mice that only received EtOH vehicle or the mice that received anti-TNFα antibodies post TNBS enema (Figure 4b). Areas of immune cell infiltration and lymphoid follicles in the submucosa were also larger in the colonic tissue of mice treated with TNBS enema alone as compared with the group that received 0.4 mg of curli (Figure 4c).

Faecal matter from each mice in each group was collected at day 2 or day 6 post TNBS enema and scored according to a previously described stool scoring system that looked for stool consistency.^59^ Mice that received only TNBS enema had higher stool scores in their faecal matter than mice that received curli (0.1 or 0.4 mg) or anti-TNFα antibody treatments day 2 post TNBS enema (Figure 4d). No differences were observed at day 6 between any of the groups (Supplementary Figure S7).

**Figure 5:**
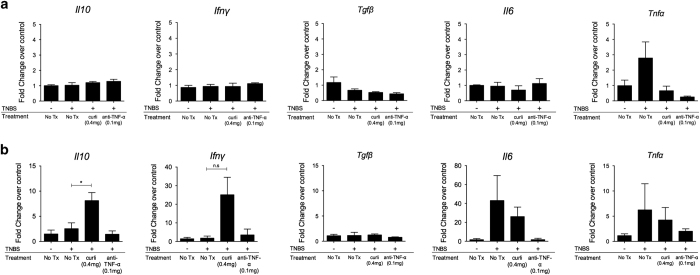
Treatment with curli fibres induce *Il10* expression in the colons of colitic mice. Colonic tissues were harvested from colitic mice at day 3 (**a**) and 6 (**b**) post TNBS enema (*n*=6). Real-time PCR analysis was performed for the expression of *Il10*, *Ifn-γ*, *Tgf-β, Il6* and *Tnfα.* Elevated *Il10* expression was observed at day 6 in the colon (*P<0.05); *P*>0.05 (NS). NS, not significant; TNBS, 2,4,6-trinitrobenzene sulphonic acid; Tx, treatment.

Following euthanasia of mice at day 3 or day 6 post TNBS induction, we assayed for the expression of various cytokines in distal colonic tissue via RT-PCR. At day 3 after TNBS treatment, we saw no significant differences in *Il10, Ifng, Tgfb, Tnfa or Il6* expression between the different treatment groups (Figure 5a). At day 6 after TNBS treatment, expression of *Il6* and *Tnfa* messenger RNA were increased after TNBS treatment. Interestingly, only administration of curli hightened transcript levels of *Il10* by day 6 post TNBS treatment (Figure 5b). An elevated level of *Ifng* detected in the intestinal mucosa of mice receiving curli (Figure 5b) did not correlate with the severity of inflammation detected by histopathology. Collectively, these results suggest to us that IL-10 may be one of the critical players in dampening of the inflammatory responses in the mice that received curli fibre treatment following TNBS enema.

## Discussion

Systemic administration of the TLR5 ligand flagellin enhances mucosal barrier function by inducing gene expression in the intestinal epithelium.^54^ Similarly, here we show that systemic administration of curli, a bacterial amyloid that stimulates the TLR2/1 receptor complex,^15,29,30^ induced *Il10* transcription in the intestinal mucosa. Furthermore, *ex vivo* exposure of intestinal cells to curli resulted in TLR2-dependent IL-10 production. As amyloids are generally resistant to proteolysis,^18^ we investigated whether expression of this anti-inflammatory cytokine could also be induced after oral administration of curli. Remarkably, oral administration of curli elevated *Il10* transcript levels in the intestinal mucosa of mice with hapten-induced colitis, whereas no significant increase was observed in conventional mice. These data suggested that induction of *Il10* expression by curli is most pronounced when this ligand can enter tissue, either after intraperitoneal injection or when epithelial integrity is impaired during colitis. Similar observations have been made with polysaccharide A (PSA), a TLR2 ligand expressed by commensal *Bacteroides fragilis* that induces a TLR2-dependent generation of IL-10-producing FoxP3+ T-regulatory cells.^60,61^ Although *csg* gene cluster, that encodes curli fibres, is found in members of *Enterobacteriaceae*, the homologues of *csg* genes were recently determined in the members of Bacteroidetes, Proteobacteria, Firmicutes and Thermodesulfobacteria.^25^ As Bacteroidetes, Proteobacteria and Firmicutes are the most abundant bacteria found in gut microbiota,^62^ these recent studies suggest that numerous bacterial species may express amyloids in the gut. Consistent with this idea, amyloids were recently detected in the biofilms of Bacteriodetes and Firmicutes isolated from sludge wastewater treatment plants.^63^ Nonetheless, further detailed analyses are needed to reveal the abundance of amyloids in the gut communities. In addition, determining the cell populations responsible for IL-10 production in response to curli fibres, e.g., FoxP3+ T-regulatory cells or regulatory monocytes, would reveal novel information in host–microbiota interactions.

Given the importance of IL-10 in acting as an anti-inflammatory molecule dampening inflammation and controlling infections,^64,65^ we explored the potential of curli fibres to reduce intestinal inflammation. Here, we show that single intragastric treatment with curli fibres ameliorates TNBS-induced colitis pathology to a similar degree as treatment with anti-TNFα antibody. EcN has been shown to reduce the severity of colitis and elicit a TLR2-dependent production of IL-10 in the gut.^49,50^ However, it is not clear whether the growth conditions used in these studies promoted the expression of curli fibres in EcN.

Epithelial cells and LP macrophages are known to be unresponsive to LPS to avoid excessive inflammation that would be triggered by the members of the microbiota,^66,67^ thus other ligands in *E. coli* become important in the gut mucosa. Even though purified lipoproteins from *E. coli* also trigger TLR2 activation, when curli fibres are present, it was reported that they are the predominant TLR2/TLR1 ligand on *E. coli*, probably because the lipoproteins remain buried in the outer membrane, whereas bacterial amyloids are secreted to the cell surface.^15^ Our results showing that the curli fibres from EcN trigger IL-10 production via TLR2 activation suggest that the EcN fibres may be expressed in the GI tract regardless of the *in vitro* growth conditions. However, further studies are needed to test whether curli fibres may be one factor that contributes to the immunomodulatory effects of EcN in the gut.

Recently, it was determined that curli fibres complex with extracellular DNA in biofilms. Even though curli is subjected to DNAse and RNase treatment during purification, extracellular DNA embedded into the amyloid structure remains intact. Thus, it is possible that the extracellular DNA associated with the purified curli preparations may also be stimulating the immune system via the cytosolic DNA sensors leading to immune homeostasis in the gut.^68^ TLR9 recognises bacterial double-stranded DNA.^51^ Polymorphisms in TLR2, NOD2, NLRP3 and TLR9 have all been identified as risk factors for developing inflammatory bowel disease.^69–71^ Thus, the success of curli treatment in colitis may be owing to the activation of multiple receptors with this bacterial component including TLR2 and NLRP3 and possibly TLR9. However, detailed analysis will reveal the pathways and receptors activated by oral curli administration in the gut.

Oral administration of curli fibres represents an exciting new strategy to ameliorate intestinal inflammation. Nevertheless, the translocation of curli fibres from the gut into the systemic circulation must carefully be monitored as amyloids are associated with many complex human diseases and considered pathogenic.^72^ It has previously been determined that systemic presence of curli fibres may lead to the production of pro-inflammatory cytokines, nitric oxide and autoantibodies.^68,73,74^ However, it is not known whether significant amounts of lumenal curli would cross into the LP and the systemic sites during colitis as phagocytes are capable of digesting and clearing amyloids. Overall, the interaction of gut mucosa with bacterial biofilm material is an intriguing line of research that needs to be explored further.
