# Supplementary material for: Biofilm-associated bacterial amyloids dampen inflammation in the gut: oral treatment with curli fibres reduces the severity of hapten-induced colitis in mice
Source: NPJ Biofilms Microbiomes. 2015 Oct 14;1:15019–. doi: 10.1038/npjbiofilms.2015.19 (PMC4739805; doi:10.1038/npjbiofilms.2015.19)
Supplement: Supplemental Information [file npjbiofilms201519-s1.pdf]

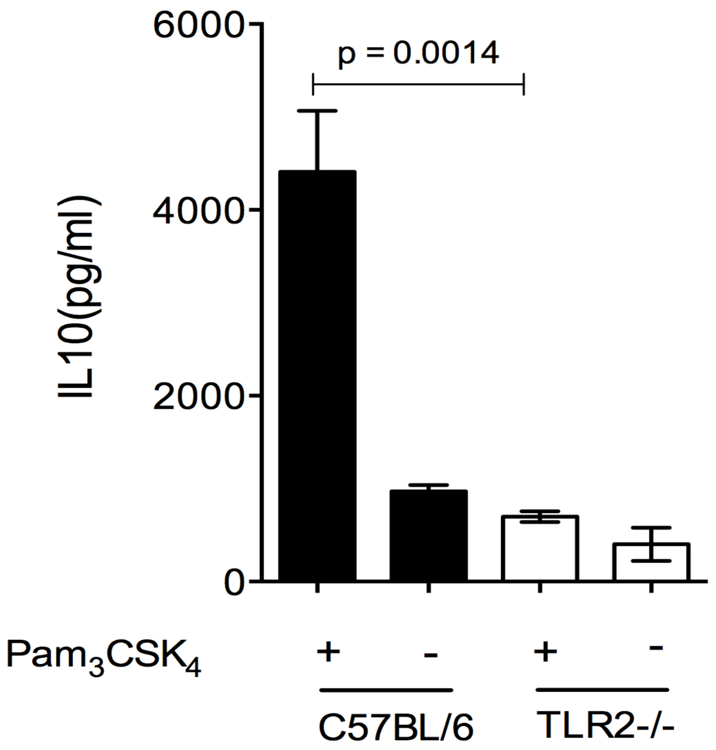

Figure S1 – Synthetic TLR2/1 ligand induces IL10 production from bone marrow-derived macrophages. Bone marrow-derived macrophages (BMDMs) from 6-8 week old female C57BL/6 and TLR2-/- mice were stimulated with 0.2μg/ml triacylated lipoprotein Pam3CSK4 for 24h. Supernatants were collected at this time point. Protein levels of IL-10 were measured in supernatants using ELISA.

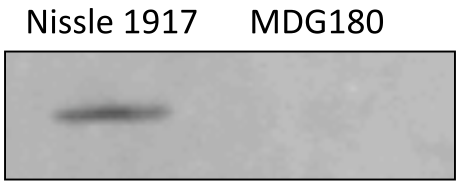

Figure S2 – *E. coli* Nissle 1917 expresses curli fibers. Western Blot analysis of curli production in Nissle 1917 and its ΔcsgA mutant, MDG180, using polyclonal anti-CsgA antibodies.

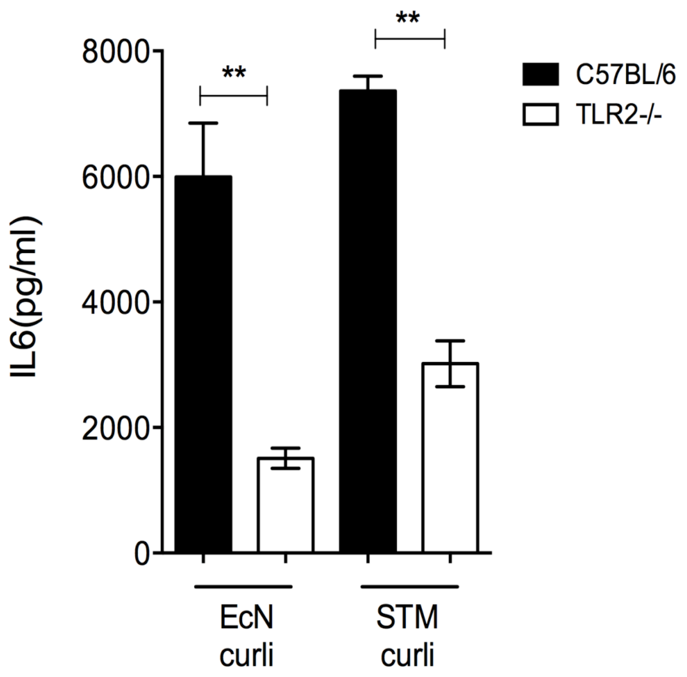

Figure S3 - Curli fibers from commensal and pathogenic bacteria induce IL-6 from bone marrow-derived macrophages in a TLR2-dependent manner. Incubation of 5μg/ml of curli extracted from a human commensal *E.coli* Nissle 1917 (EcN) or from *S*. Typhimurium elicited IL-6 to a similar extent from BMDMs in a TLR2-dependent manner.

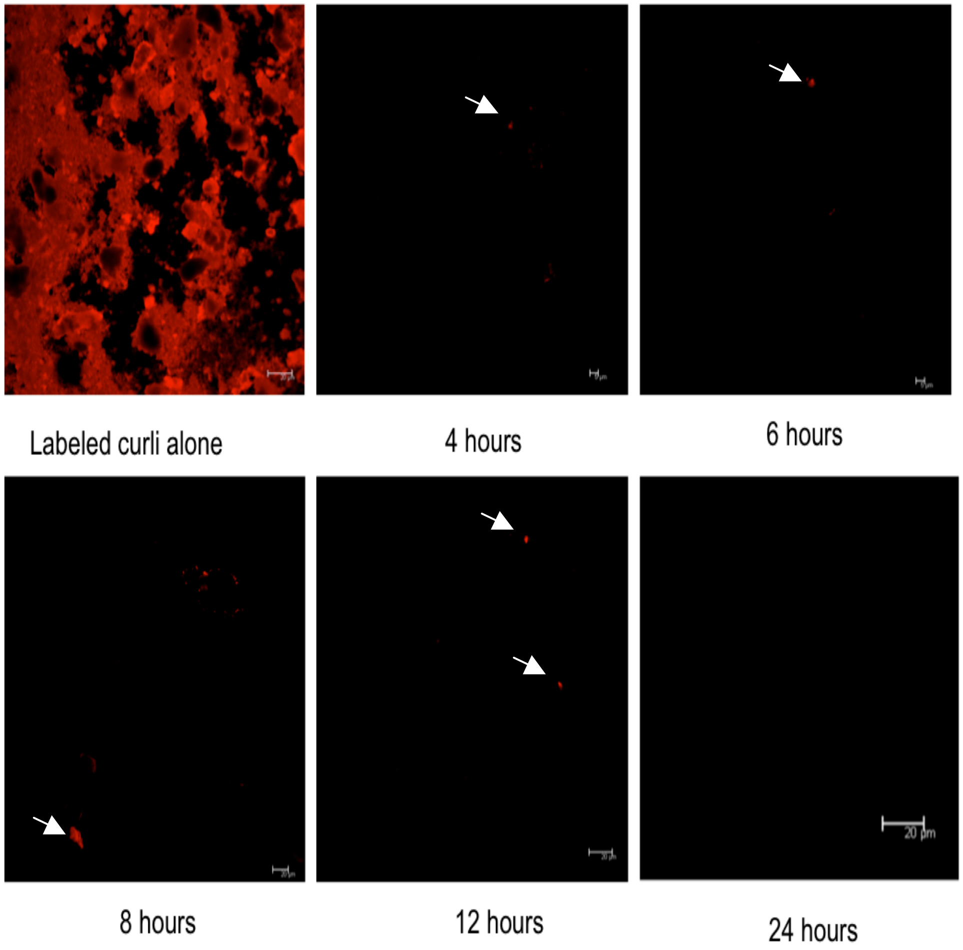

Figure S4 – Curli fiber movement through GI tract

Purified curli fibers from STM were labeled with the Dylight® 633. 8-week-old female Balb/c mice were then administered 0.1mg curli fibers via oral gavage. 2-3 fecal pellets were collected from each mice at the indicated time-points. The fecal pellets were resuspended in PBS and then imaged using Leica SP5 confocal microscope. Data is representative of two independent experiments.

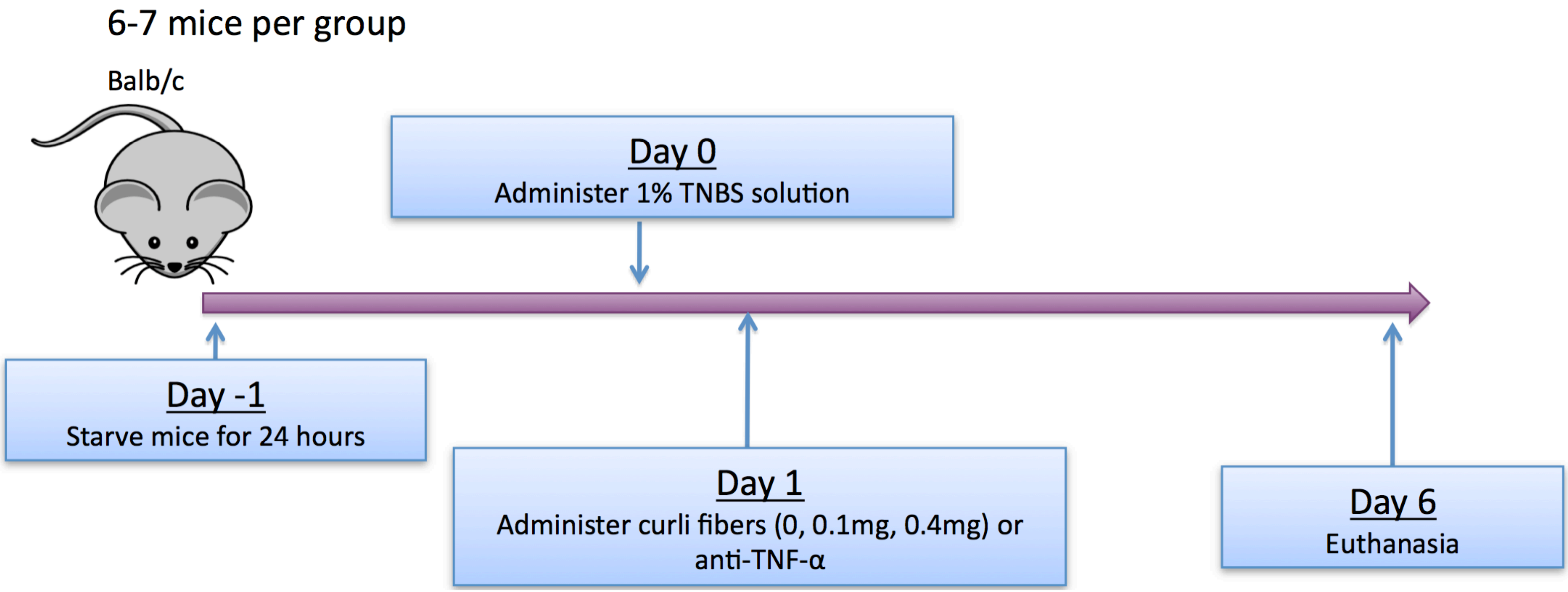

Figure S5 – Experimental time course for TNBS colitis induction in 6-8 week old female Balb/c mice. 6-8 week old female Balb/c mice were starved for 24 hours prior to administration of 1% TNBS intrarectally. One day after the TNBS enema, mice were treated with 0.1mg or 0.4mg curli fibers intragastrically or anti-TNF-α intraperitoneally. Mice were euthanized on day 6 following the administration of TNBS.

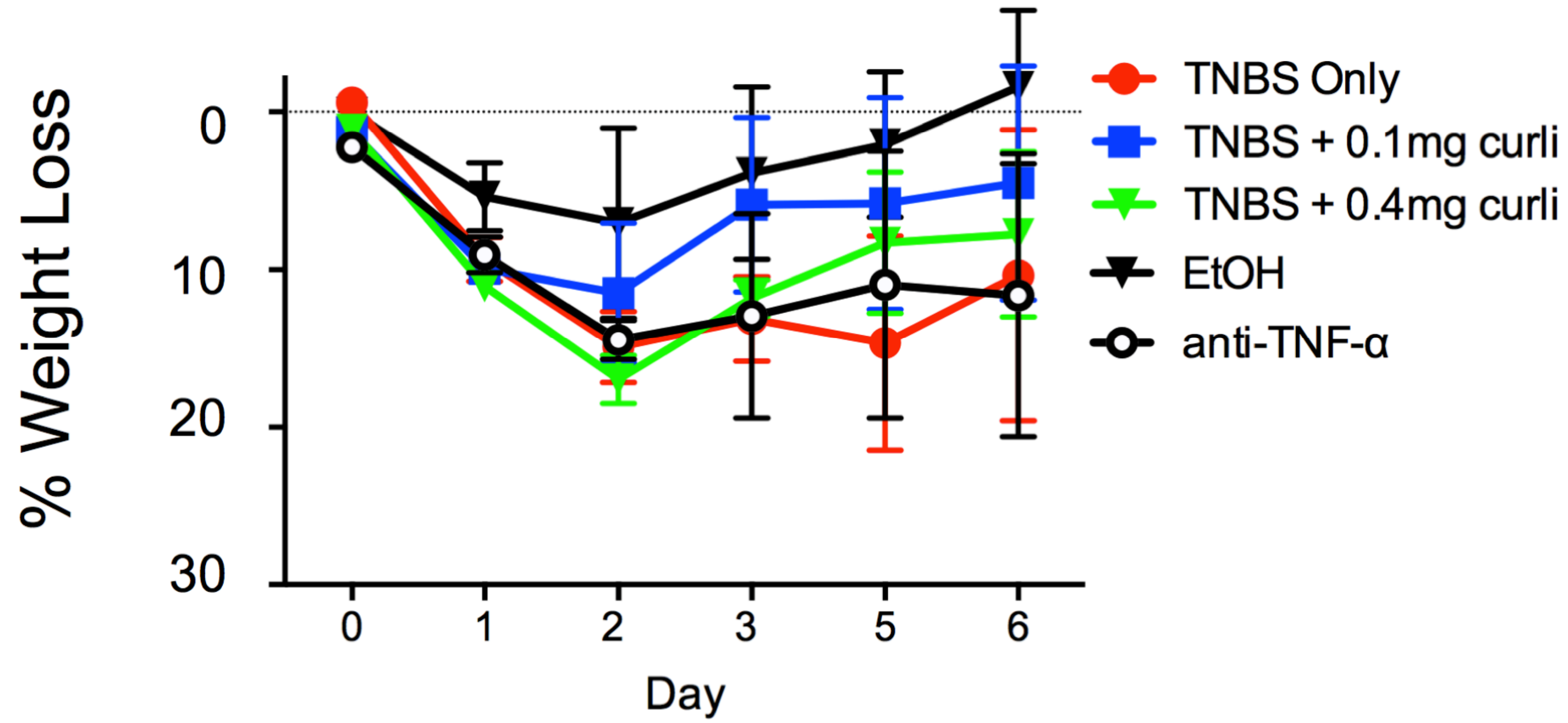

Figure S6 – Effect of curli fibers on TNBS colitis-induced weight loss. A single treatment of colitic mice with curli fibers rescued mice from TNBS colitis-induced weight loss.

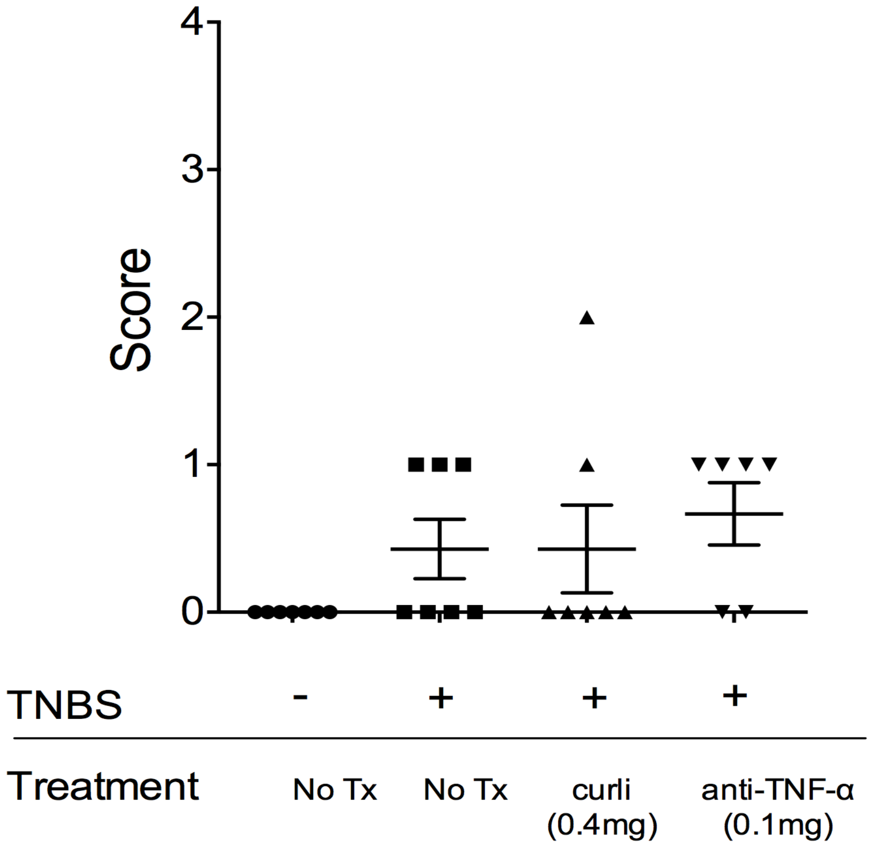

Figure S7 – Stool consistency scores in colitic mice at day 6 post-TNBS colitis induction. No significant differences were observed in stool consistency between mice in the different experimental groups at day 6 following induction of colitis.
